# Advancing opioid stewardship in low-middle-income countries: challenges and opportunities

**DOI:** 10.1080/20523211.2024.2345219

**Published:** 2024-06-05

**Authors:** Rojita Jadhari, Nabin Pathak, Rajeev Shrestha, Sunil Shrestha, Bhuvan KC, Siew Hua Gan, Vibhu Paudyal

**Affiliations:** a Drug Discovery and Development, Faculty of Pharmacy, Uppsala University, Uppsala, Sweden; b Drug Information Unit and Pharmacovigilance Cell, Department of Pharmacy, Hetauda Hospital, Madan Bhandari Academy of Health Sciences, Bagmati Province, Makwanpur, Hetauda, Nepal; c Department of Pharmacy and Clinical Pharmacology, Madan Bhandari Academy of Health Sciences, Bagmati Province, Makwanpur, Hetauda, Nepal; d Palliative Care and Chronic Disease, INF Nepal Green Pastures Hospital and Rehabilitation Centre, Pokhara, Nepal; e School of Pharmacy, Monash University Malaysia, Bandar Sunway, Malaysia; f School of Clinical Sciences, Queensland University of Technology, Brisbane, Australia; g College of Public Health, Medical and Veterinary Sciences, James Cook University, Townsville, Australia; h Florence Nightingale Faculty of Nursing, Midwifery and Palliative Care, King's College London, London, United Kingdom; i School of Pharmacy, College of Medical and Dental Sciences, University of Birmingham, Birmingham, United Kingdom

**Keywords:** Opioids, Opioid stewardship, Pharmacist, Low-and middle-income countries

## Abstract

The increased utilization of opioids in low– and middle-income countries (LMICs) presents a growing threat of opioid-related abuse, misuse and diversion. Pharmacists, as integral members of healthcare teams, are responsible for dispensing and monitoring opioid use and hold a pivotal role in opioid stewardship within LMICs. This commentary describes the potential and multifaceted roles of pharmacists in opioid stewardship in resource-constrained settings and highlights appropriate strategies for promoting responsible opioid utilization.

Opioid stewardship involves prioritising evidence-based prescribing, management and practices for pain management. It includes measures such as incorporating prescription drug monitoring programmes for appropriate opioid prescription, implementing safe disposal through drug take-back programmes, promoting non-opioid pain management, addressing the opioid addiction stigma, tapering opioid dose, educating patients and caregivers, establishing drug information centers, providing rehabilitative services and integrating collaboration with communities and experts.

The combined difficulties of restricted access to healthcare resources and services coupled with low levels of literacy worsen the susceptibility to opioid abuse, misuse, and diversion in LMICs. Early detection, assessment and implementation of interventions to optimise opioid use are imperative for ensuring safe and effective opioid utilization, thereby mitigating the risks of overdose and addiction. The involvement of pharmacists in promoting safe and effective opioid utilization through education, monitoring, collaboration, and policy advocacy serves as a critical component in bridging existing gaps in opioid stewardship within LMICs.

## Introduction

Although poppy-derived opioids are widely used for pain, they pose addiction and side effects risk (Norn et al., 2005; Trang et al., 2015; Krashin et al., 2013; Benyamin et al., 2008). Certain opioids such as heroin, morphine, codeine, fentanyl, methadone and tramadol can be misused for their euphoric effects (Pathan & Williams, 2012; Rosenblum et al., 2008), making it a global issue (National Academies of Sciences E, Medicine, 2017). In 1986, the widespread marketing and campaigning for opioid use to treat non-cancer pain triggered a significant shift in the opioid epidemic (Kolodny et al., 2015), causing a notable increase in the death toll as a result of opioid overdose (Chisholm-Burns et al., 2019). As per the World Health Organization (WHO), 600,000 deaths globally were due to drug use in 2019, among which 80% of deaths were related to opioids, with 25% of fatalities caused by opioid overdose (WHO, 2023). As of July 2023, approximately 3 million Americans and 16 million individuals suffer from opioid use disorder (OUD) globally (Azadfard et al., 2021). It has been reported that inadequate knowledge and promotion contributed to the opioid epidemic in the United States (US) and Canada (Brown & Morgan, 2019). High-income countries (HICs) like the US and Canada face a high burden of opioid overdose deaths, necessitating strategies for prevention and treatment (Brown & Morgan, 2019). Similarly, the data provided by the National Center for Health Statistics from 1991 to 2021 indicates an increasing trend in opioid overdose deaths in the US, reaching over 80,000 in 2021 (National Institute on Drug Abuse, 2023).

The newly proposed definition of opioid stewardship programmes (OSPs) includes evidence-based guidelines, policies, person-centred practices and research to promote rational prescribing, use and deprescribing of opioids for managing pain and specific health conditions (Shrestha et al., 2023). Deprescribing refers to stopping the drug or reducing its dose when the drugs’ use is no longer necessary as it may pose harm (Wu et al., 2023). OSPs optimizes the treatment by maximising clinical benefits for the patients and the wider society, minimising adverse consequences, including opioid-related abuse, misuse and diversion. Effective patient-provider communication involving patients and/or their caregivers in decision-making is key to implementation of any OSPs by considering evidence-based outcomes that matter to patients. OSPs also focus on safe procurement, storage and disposal practices (Shrestha et al., 2023). Many opioid patients tend to misuse or develop OUD (8–12% and 21–29%, respectively) due to opioid prescription (Vowles et al., 2015). The situation is compounded when individuals, regardless of age, may transition from medical to non-medical opioid use (McCabe et al., 2017), highlighting the broad scope of vulnerability. Therefore, to curb dependency, the Drug Enforcement Administration (DEA) reduced opioid manufacturing by 20% in 2018, which raises some concerns about pain management (Bruera Eduardo, 2018). In summary, regulating opioid use remains a challenge in both HICs and low-middle-income countries (LMICs).

Previous reports refer to suboptimal pain management and provision of emotional support to patients in LMICs, highlighting the need for more effective pain management interventions and improved availability of opioid analgesics (Sasaki et al. (2017). Limited access to pain management and addiction treatment services exacerbates the challenges, individuals with OUD face concerning LMICs.

## Role of pharmacist in opioid stewardship

Recent systematic reviews and meta-analyses have shown that pharmacists have the potential to effectively manage pain through opioid optimization (Shrestha, Ayesha et al., 2024; Shrestha, Kc et al., 2022; Thapa et al., 2021). Pharmacists play a crucial role in responsible monitoring and the use of opioids to ensure safe and effective pain management while minimising harm such as addiction and overdose. Potential for improved opioid stewardship by pharmacists through active interventions such as medication reviews and pain assessment has been reported too (Gondora et al., 2022). Assessing medication regimens, drug–drug interaction, dosage adjustment, and educational programs have shown to be effective in reducing pain intensity and the mean morphine equivalent doses (Cid et al., 2023; Shrestha et al., 2022; Veettil et al., 2022). Pharmacists’ roles have been found to be effective in identifying drug-related problems such as through the use of long-acting opioids (instead of the shorter-acting ones) and introducing the use of long-acting opioids to treat naïve patients, as well as reducing adverse effects related to opioid medications (Cid et al., 2023).

Pharmacists in HICs contribute to patient screening, risk stratification, education and outreach for better pain management, medication therapy management (MTM), safe storage, disposal, distribution of naloxone and referrals to addiction treatment (Chisholm-Burns et al., 2019; Dolovich et al., 2019). Their roles have been reported to be key to substance abuse prevention and treatment programs, and they are known to collaborate with authorities as well as healthcare professionals on detoxification protocols and opioid overdose treatment (ASHP, 2014). Pharmacists have supported the provision of medication-assisted treatment with methadone, buprenorphine and naltrexone, which have the potential to reduce opioid addiction and mortality (Chen et al., 2021; Connery, 2015; Schwartz et al., 2013). However, some studies suggest that pharmacists in HICs may lack comprehensive clinical guidance on opioid-related interventions, highlighting the need for evidence-based support and collaborative practice models (Webb et al., 2021). Indeed, community pharmacists in HICs have a vital role in communicating with patients, reinforcing proper opioid use and preventing opioid abuse, utilizing their knowledge of drug monitoring programs, safe storage and disposal, risk assessment and provision of naloxone to people at risk of opioid overdose (Gregory & Gregory, 2020). Their expertise can significantly contribute to opioid stewardship for improved patient outcomes. With the emerging opioid-related abuse, misuse and diversion in LMICs, the opioid stewardship measures of HICs can be adopted by pharmacists in LMICs with a collaborative approach to tackle opioid-related issues. Provision of education to patients concerning optimal use, evidence based and potential for misuse and harm, and involving patients in decision making are some of the patient centred activities relevant for pharmacists to undertake.

## Opioid stewardship strategies

**Figure 1.**
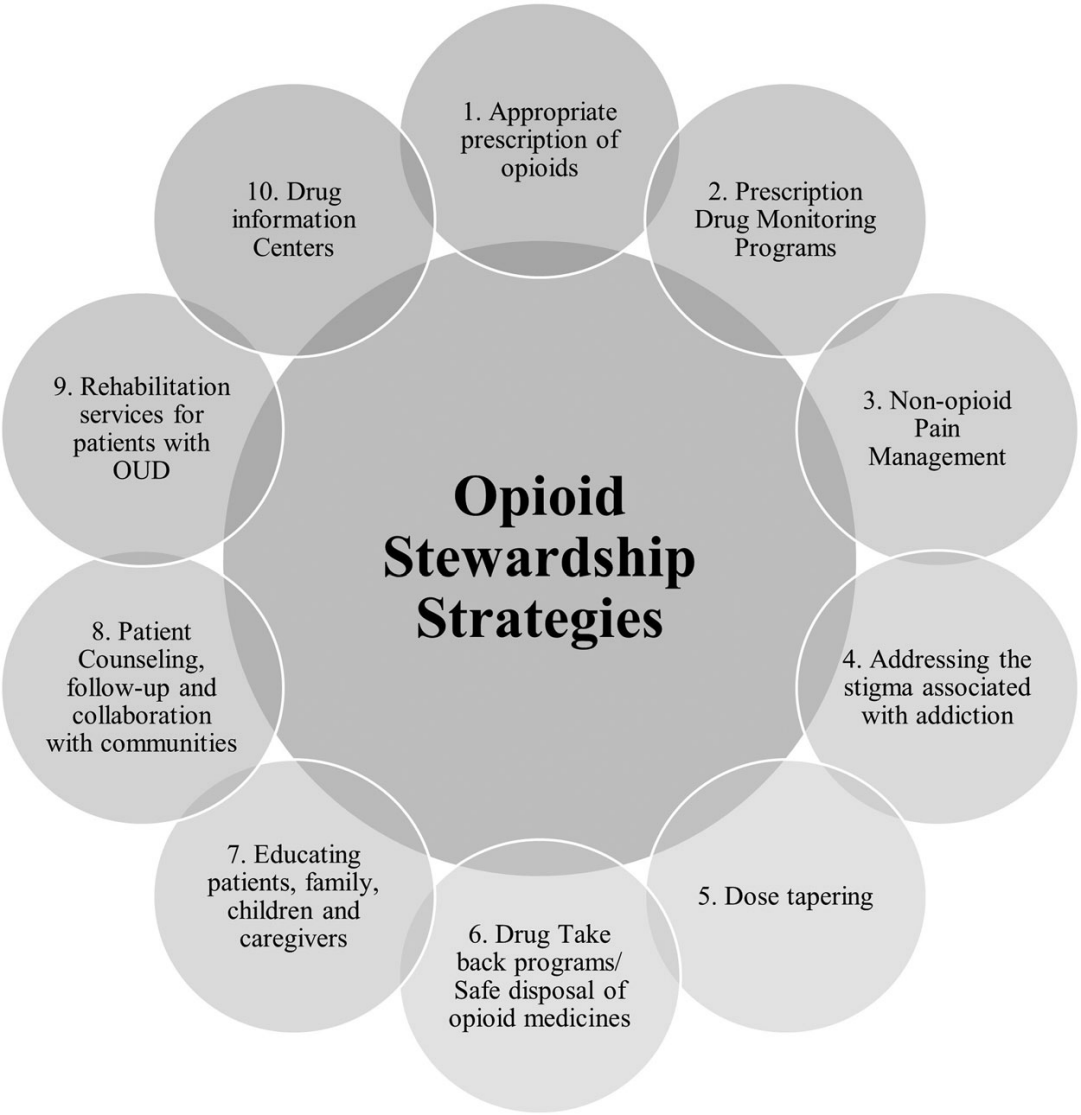
Opioid stewardship strategies.

OSPs provides a framework for improving opioid prescribing practices, OUD treatment, patient education and technology utilization to monitor opioid misuse (Figure 1) (American Hospital Association, 2017).

### Appropriate prescription of opioids

Appropriate prescription practices involve prescribing the lowest effective opioid dose for the shortest duration, utilising non-opioid alternatives for pain management and adhering to clinical guidelines. This approach promotes safe and effective chronic pain management while preventing misuse (American Hospital Association, 2017; Dowell et al., 2022). In addition, the prescription limitation is effective in supporting opioid stewardship and preventing overprescribing (Gregory & Gregory, 2020). To combat overprescribing, many states in the US and some healthcare organisations have implemented prescription limits, typically ranging from 3 to 14 days for initial opioid prescriptions (Dowell et al., 2022). These limitations and close pharmacist monitoring help track patient progress and identify misuse or abuse. Similarly, the introduction of novel drug delivery systems on opioid medications can help provide slow or sustained release effects along with diminished opioid-related adverse events and opioid abuse potential (Soltani & Pardakhty, 2016).

Interdisciplinary communication is key in optimizing treatment. Limited communication between prescribers and pharmacists can lead to suboptimal care. Good communication requires active listening, documentation, and asking relevant questions in a friendly manner. Pharmacists can improve communications with prescribers by regularly discussing opioid use, their potential misuse, and the formation of consensus guidelines. Furthermore, drug and therapeutic committee participation may facilitate effective communication among pharmacists and other healthcare professionals.

### Incorporating Prescription Drug Monitoring Programs (PDMPs)

The roles described in HICs also include the incorporation of PDMPs to address opioid misuse and over-prescribing. Research shows that the implementation of doctor-shopping laws and pain clinic regulations has led to a reduction in prescription opioid overdose deaths by 8.5% and 9.6%, respectively (Popovici et al., 2018). PDMPs are crucial in controlling opioid misuse and overdose deaths (Delcher et al., 2020). In New York City, mandatory PDMPs laws reduced ‘doctor shopping’ and opioid misuse (Bachhuber et al., 2019). Unfortunately, LMICs often lack the implementation of such monitoring programmes, leaving opioid consumers vulnerable to addiction. However, pharmacists in LMICs can leverage PDMPs to identify patients at risk for OUD and to provide appropriate interventions, including treatment referrals and support for opioid tapering. Despite PDMPs’ significant role in OSPs, their practicality may be an issue within LMICs since some prescribers and pharmacists may not closely monitor patients’ medication to promote medicine prescribing (Miller & Goodman, 2016). Profit-oriented healthcare practice can be a key challenge to treatment optimization, roles and responsibilities of prescribers and pharmacists along with incorporating training and awareness programs (Babar, 2021).

### Alternative method of pain management (non-opioid pain management)

Careful consideration of the risks and benefits of opioid treatment is crucial for effective pain management to prevent dependency. Abuse, dependence and overdose are associated with long-term opioid use for chronic pain (Bicket et al., 2019). Thus, non-opioid treatments to manage persistent and recurring chronic pain are required to avoid such unwanted effects. Evidence-based practices with the integrated close input of pharmacists and other healthcare personnel have been reported to effectively reduce opioid use and effective pain management in post-surgery patients with non-opioid analgesic techniques (Bicket et al., 2019). Additionally, non-opioid analgesics such as acetaminophen, non-steroidal anti-inflammatory drugs (NSAIDs) such as ibuprofen, diclofenac, naproxen, cyclo-oxygenase-2 (COX-2) inhibitors, aspirin, anticonvulsants (gabapentin and pregabalin), antidepressants (amitriptyline and duloxetine) and topical agents such as lidocaine and capsaicin available both over the counter or via prescription can be recommended (NEJM Knowledge+ Team, 2020). However, it is essential to understand that non-opioid analgesics are also associated with potential side effects and must be used under a licensed prescriber’s guidance and observation. For chronic pain management, many individuals are turning to complementary health approaches, including acupuncture, massage, meditation, yoga and the use of natural products and supplements (Nahin et al., 2016). However, the evidence base around complementary and alternative practices is less understood than conventional medical practices.

Furthermore, patients should be encouraged to follow non-pharmacological methods to ease their pain rather than being solely dependent on opioid medications to manage chronic pain. Additionally, patients should be encouraged to use non-opioid analgesics and evidence-based complementary approaches for chronic pain management rather than solely relying on opioids.

### Addressing the stigma associated with addiction (opioid dependence)

Due to low health literacy, the stigma associated with OUD remains deeply rooted in many patients (Cheetham et al., 2022). OUD tends to drive away individuals dependent on opioid use from seeking help to avoid being marginalised or neglected by society. The lack of education and awareness regarding the rational use of opioids, the withdrawal symptoms and adverse effects further influence the consequences. OUD is associated with several adverse health outcomes, such as opioid overdose, suicide, accidental injuries, addiction, and transmission of infectious diseases like human immunodeficiency virus (HIV). The disease stigma can originate from various sources, including misunderstandings about drug addiction, the fear of legal consequences as well as negative stereotypes about individuals who use opioids (Degenhardt et al., 2019), which may lead to under-treatment of OUD. Therefore, understanding the stigma behind OUD is crucial to normalising the patient for seeking help and ensuring they get the necessary support and treatment. This goal is attainable in LMICs through pharmacists’ comprehensive medicinal counselling. Pharmacist role is critical in promoting education on overdose treatment and fostering a supportive environment for individuals struggling with OUD.

### Dose tapering

Dose tapering is the gradual reduction of opioid medication dosage, typically undertaken when the use of opioids is no longer necessary, rise of adverse effects or a risk of opioid dependence. This approach is particularly crucial for patients managing cancer or chronic non-cancer pain. Abrupt discontinuation of opioid’s use can lead to physical dependency and withdrawal symptoms among long-term users. Therefore, a slow tapering process is recommended to minimize these risks and comfort patients. However, it is essential to understand that the use of opioids in most terminal stages of cancer is usually not associated with addiction (Opioid Use Disorder, 2024); hence, diminishing its dosage is not an option, as the primary ethical concern in end-stage cancer patients is still to alleviate pain. Healthcare professionals, including pharmacists, should assess the necessity of dose tapering based on a patient’s requirements and circumstances. Many clinicians adhere to the USA Department of Health and Human Services guidelines to safely taper off opioids for patients on long-term usage (HHS, 2019). This structured approach ensures that dose tapering is conducted safely and effectively, minimizing potential risks and promoting the patient’s well-being.

### Safe disposal of opioid medicines/drug take-back programmes

A study conducted on drug take-back programmes and permanent drug donation boxes in Kentucky reported that from the 802 lbs of collected medications, 25 lbs (3%) were controlled substances, of which 40% were opioid analgesics (Egan et al., 2017). Hence, implementing such drug take-back programmes is crucial for safe disposal of unused and expired drugs since it helps prevent accidental poisoning, overdosing and illicit drug use. However, a problem may still be the use of illegal opioids. In such cases, clear transparency in opioid manufacturing, prescribing, sales, recording and distribution, and take-back channels must be maintained and monitored by the governing bodies so that any illegal distribution can be discouraged. PDMPs generally associate themselves with law enforcement agencies. Some examples of such programmes involved in drug disposals are the ‘Operation of a Medicine Cabinet’, ‘Take-back Your Meds’, and ‘the American Medicine Chest Challenge’ (Rural Health Information Hub, 2020). Establishing such programmes in LMICs will require substantial government support and collaboration with local community groups. Education and awareness campaigns are vital in promoting participation in these programmes.

Furthermore, conducting comprehensive research is necessary to evaluate the effectiveness of these strategies. Following HICs, collaboration between national drug regulatory authorities and ministries responsible for opioid regulations in LMICs, together with the police, drug stores, and local pharmacies, is crucial in managing unused, unwanted and expired medication for safe disposal as well as discouraging illegal opioid distribution. A pilot study to confirm the implementation of the drug take-back concept within the communities of an LMIC concluded that the participants were eager to support the program if implemented; however, the major challenge could be an allocation of budget from the government (Sapkota et al., 2022).

Moreover, to address the opioid crisis, pharmacists can take a comprehensive approach by focusing on appropriate opioid prescriptions, implementing opioid take-back practices and ensuring the safe disposal of opioids. The DEA creates awareness and provides a location for twice yearly disposal of unused and unwanted medicine (Drug Enforcement Administration, 2020). The said approach is essential in both community and hospital settings and is aimed at preventing opioid misuse while promoting community well-being.

### Educating patients, family, children, and caregivers

Awareness about the risk that comes with opioid consumption must be communicated to the patient, family, and caregiver. Pharmacists must appropriately counsel these groups to ensure they are well-informed and aware of OUD. Active participation of patients in their treatment plan and compliance is critical to health outcomes. Pharmacists act as an inter-chain between physician and patient in bridging the communication gap. Hence, they should communicate with patients regarding opioid medication counselling that involves confirming whether the medication prescribed is an opioid or not, its overdose and dependency risks, along with opioid safety, which consists of the use of naloxone in opioid overdose cases (Thakur et al., 2021). On the other hand, low health literacy prevents patients from understanding the health consequences of inappropriate healthcare decisions (Budhathoki et al., 2017). Similarly, it is imperative to teach students and children about the harms of opioids, for which the development of a standard curriculum focusing on opioid use and OUD can be performed by taking an example from HICs (Yereth Rosen, 2020).

### Patient counseling, follow-up, and collaboration with the communities

Dispensing of opioid medications within the hospital and in the community pharmacy settings must include comprehensive counselling on the risks associated with opioid overdose. Patients should be informed about the importance of seeking medical assistance in case of overdose, adverse effects, or withdrawal symptoms. Before dispensing the prescribed amount of medication, pharmacists should review the patient’s medical history and ensure their understanding of proper drug use and dosage regimen. In addition, pharmacists should frequently interact with patients, which helps establish a robust patient-pharmacist relationship. The bond and trust enable patients’ reliance on pharmacist’s advice.

By effectively counselling patients on the risks associated with unnecessary opioid use and maintaining regular follow-ups, pharmacists can play a vital role in minimizing the likelihood of opioid-related harm (Bach & Hartung, 2019). However, it is crucial to emphasize pharmacists’ training in patient counselling to maintain healthy communication with OUD patients.

In addressing the opioid crisis, community pharmacists play a vital role in expanding drug use disorder treatment services, equipping first responders with naloxone, integrating physical and behavioural healthcare, collaborating with law enforcement to facilitate treatment access, funding public education programmes, educating community clinicians and participating in drug take-back initiatives. Combating the opioid epidemic requires a multidisciplinary approach and close collaboration between clinicians and communities. Additionally, having coordinated community responses that involve local political leaders, healthcare providers, law enforcement and state and local healthcare centres, is essential to curb the opioid epidemic effectively (American Hospital Association, 2017). LMICs community pharmacies can take insights and learnings from HICs in educating patients about OUD and the risks associated with opioids.

### Rehabilitation services for patients with OUD

Knowing when to refer a patient to addiction/rehabilitation services is crucial. Suppose the patient shows opioid withdrawal symptoms and is under opioid dependence. Pharmacists should interact with prescribers and provide the necessary rehabilitation services or refer patients to accessible addiction/rehabilitation care centres (Gregory & Gregory, 2020). Pharmacists can access literature to garner pertinent information and render advice to those fighting addiction to make a difference. Similarly, pharmacists can also engage in medical reviews with the available patient history to help in recommending better medication therapy that can be less addictive and also help in delivering and dispensing life-saving drugs, including naloxone, methadone or buprenorphine to those at risk of overdosing and is associated with OUD (Bach & Hartung, 2019; The Frontline in the Fight Against Addiction, 2024). Early intervention and access to medicine-assisted treatment for individuals struggling with opioid addiction can help in improving health outcomes (Connery, 2015). Drawing from practices in HICs, the rehabilitation centres in LMICs may benefit from hiring pharmacists to help patients with OUD achieve abstinence or to manage withdrawal symptoms.

### Drug information centers

To successfully implement the objective of OSPs, healthcare professionals, patients, caregivers, and family members must comprehensively understand appropriate opioid use, associated risks and safety measures. DICs play a crucial role in bridging the knowledge gap by providing evidence-based information, particularly in LMICs, where a study highlights the positive impact of DICs on enhancing patient safety by reducing medication errors (Shrestha et al., 2020). Within LMICs, DICs can be executed in federal and provincial health institutions, thus acting as an agency for medicine information. DICs mainly involve clinical pharmacists, pharmacologists and even pharmacists responsible for delivering pertinent opioid-related information and educating patients on the safe use of opioids. Additionally, DICs can also provide valuable information on the proper storage, disposal and administration of opioid medications, as well as the potential risks and benefits associated with their use. The widespread dissemination of DICs in various communities and hospital settings can contribute to addressing opioid-related abuse, misuse and diversion by raising awareness and delivering essential education to individuals.

## Opioid stewardship in LMICs

Non-communicable diseases (NCDs) account for 74% of all deaths globally, of which 77% are in LMICs (Noncommunicable diseases, 2023). NCDs, such as cancer, are the second leading cause of death globally, accounting for approximately 18.1 million new cases and 9.6 million deaths, or one in six deaths in 2018 (International Agency for Research on Cancer, 2018). Moreover, with an increasing number of NCDs and the severity of such diseases, the people in LMICs need better healthcare services, timely intervention and adequate access to life-saving medicines for palliative care.

Although HICs have made several advances in pharmacy practice by focusing on independent prescribing by pharmacists, clinical skills development and expanding pharmacy services, practices are still in the infancy in LMICs, underscoring the urgent need for improvement to implement opioid stewardship initiatives effectively (Babar, 2021). In LMICs, pharmacists can contribute further by providing education, counselling and monitoring for OUD, promoting the safe disposal of excess opioids and collaborating with other healthcare professionals to ensure appropriate opioid prescribing (an approach that includes prescribing adequate opioids through means of regular evaluation and planning followed by monitoring) in line with evidence-based guidelines and best practices. Engaging pharmacists and strengthening pharmacy practice can enhance LMICs’ capacity for effective opioid stewardship and address their unique challenges.

Establishing robust regulatory frameworks is essential in LMICs to ensure proper licensing, distribution and control of opioid medications. The framework involves implementing and monitoring enforcement mechanisms to prevent diversion and illegal trade and stringent regulations to ensure the safe and appropriate use of opioids. Additionally, capacity-building initiatives are also crucial in strengthening healthcare systems in LMICs. Conducting training programmes and providing educational opportunities for healthcare professionals, including pharmacists, can enhance their knowledge and skills in appropriate opioid prescribing, pain management and addiction treatment, thus enabling them to contribute effectively to opioid stewardship efforts.

Addressing challenges related to the supply chain management of opioid medications is another critical aspect, including tackling affordability, availability, quality assurance and distribution logistics issues. Public awareness campaigns are vital in educating communities in LMICs about the appropriate use of opioids, the potential risks associated with misuse and the available resources for pain management and addiction treatment. Such campaigns should reach a broad audience and provide accurate information to promote responsible opioid use and reduce the stigma associated with OUD.

Sharing best practices, knowledge and resources can help strengthen opioid management strategies in LMICs, leveraging the experiences and expertise of different entities to develop effective interventions. Research and data collection efforts are crucial for understanding the local opioid epidemic in LMICs. Gathering data on the patterns of opioid-related abuse, misuse and diversion and the effectiveness of interventions can inform evidence-based approaches in opioid stewardship, as the generated findings can guide policy development, programme implementation and resource allocation to address specific challenges of LMICs. International collaboration and partnerships between LMICs, HICs, organisations, and stakeholders are needed to promote opioid stewardship.

## Challenges of opioid stewardship in LMICs

**Figure 2.**
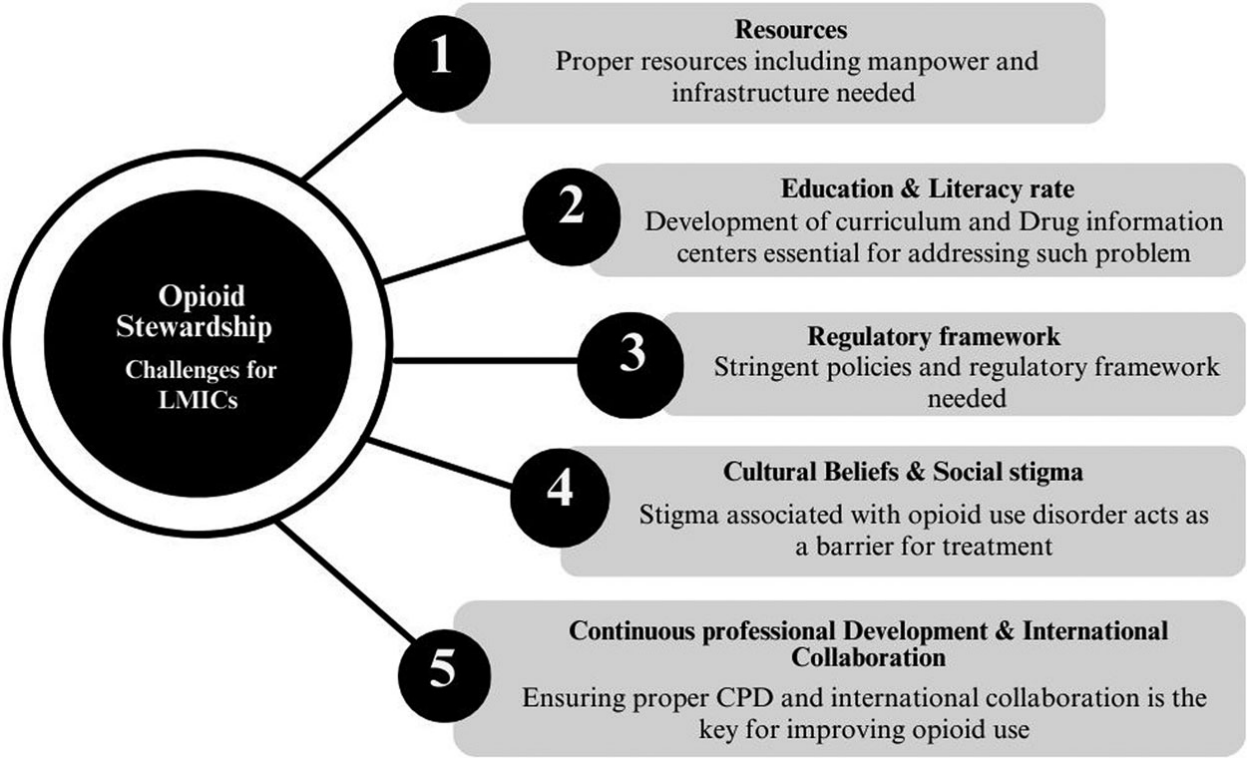
Challenges in implementing opioid stewardship in LMICs.

The challenges of opioid stewardship in LMICs are multifaceted and require careful consideration (Cernasev et al., 2021). Limited resources, including healthcare infrastructure, workforce shortages and funding constraints, hinder the implementation of comprehensive OSPs. Insufficient education and training among healthcare professionals contribute to suboptimal practices and the risk of opioid misuse (Figure 2). Inadequate regulatory frameworks and guidelines make it challenging to enforce responsible opioid use and mitigate risks. Cultural beliefs, stigma and social factors affect the acceptance and implementation of OSPs. Limited accessibility and affordability of alternative pain management options increase reliance on opioids. Additionally, co-existing basic health crises that LMICs faces divert attention and resources away from effectively managing opioid stewardship. LMICs also face a significant challenge regarding easy access to over-the-counter (OTC) use and misuse of prescription opioids due to the lack of stringent mechanisms.

Although the use of opioids in managing pain is essential, the misuse, abuse and overuse must be prevented and mitigated. The limited availability of opioids, financial instability and shortage of trained healthcare professionals contribute to further lack of comprehensive primary care services in LMICs (Harding & Higginson, 2005; Knapp et al., 2011; Shrestha & Shrestha, 2024).

A major challenge hindering adherence to OSPs is the porous borders within LMICs. The report from drivers of illicit traffickers among the border communities in Southeast Asia explains how communities located at and within the border are more vulnerable to drug-related trafficking (UNODC, 2023). Factors such as poverty and economic injustice play significant roles in the illicit trafficking of drugs. Some efforts that may ameliorate drug-related trafficking activities within porous borders includes building robust community policies, engaging community leaders and border officials, and establishing cordial relationships between border liaison officers and local police about information sharing and educational campaigns to be conducted (UNODC, 2023). Furthermore, robust regulations in procurement, production, distribution, and drug take-back programs can also contribute to reducing potential illicit trafficking. Similarly, challenges such as the absence of PDMPs, drug take-back programmes, rehabilitation services and proper education on opioids further complicate stewardship efforts. Lack of expertise, limited knowledge, and training among healthcare professionals, as well as inadequate rules and regulations in opioid prescribing and dispensing, contribute to the potential for misuse and abuse of opioids (Table 1).

**Table 1.** Challenges of opioid stewardship for LMICs.

SN	Challenges of opioid stewardship for LMICs	Comments
1	Limited resources including human resources, infrastructure	Opioid stewardship necessitates a multidisciplinary approach requiring collaboration among doctors, pharmacists and allied healthcare professionals (Shrestha et al., 2023; Smith, 2020; Uritsky et al., 2021). Adequate staffing and infrastructure are essential components for effective implementation.
2	Insufficient education and training with low literacy rates	Literacy rates play a crucial role in promoting rational drug use, and combating misuse and abuse (Eser & Çelik, 2022). Curriculum development and DICs can serve as educational avenues to enhance opioid stewardship. Primordial and primary prevention methods should be prioritised to address opioid abuse.
3	Inadequate regulatory frameworks and guidelines	Robust regulatory frameworks are imperative for driving opioid stewardship efforts, facilitating effective management of OUD, and promoting stewardship strategies. Clear guidelines streamline the management of opioid-related issues.
4	Cultural belief’s, stigma, and social factors	Cultural beliefs and social stigma associated with OUD patients often hinder sufficient medical treatment and rehabilitative services. Community education initiatives are essential to dispel myths and misconceptions surrounding OUD.
5	Limited accessibility of drugs along with their affordability	Establishing stringent procurement mechanism can address issues of drug accessibility and affordability within LMICs. Pharmacists play a significant role in ensuring equitable access to essential medications, including opioids.
6	Financial stability and funding constraints	Collaborative efforts between the Ministry of finance and relevant stakeholders are crucial in assessing and addressing opioid-related challenges before they escalate. Adequate funding is essential for implementing effective opioid stewardship initiatives.
7	Continuous Professional Development (CPD)	CPD programmes are vital for enhancing the competency of healthcare professionals and staying abreast of emerging trends in opioid stewardship. Regulatory bodies and ministries should collaborate to develop CPD programmes tailored to tackle opioid-related issues.
8	International Collaboration	Learning from the experiences of HICs and fostering collaboration between LMICs and HICs can enhance understanding and management of the opioid epidemic. Bilateral relationships, such as those between neighbouring countries like India and Nepal, can facilitate joint efforts in managing opioid use and transportation.
9	Inclusion of new ideas such as PDMPs, drug take-back programmes	Addressing issues of accessibility and affordability to opioids paves the way for the implementation of innovative solutions like PDMPs and drug take-back programmes. These initiatives are essential for preventing misuse and diversion of opioids.

## Prevention and solutions to opioid dependency in LMICs

Preventing opioid dependency is a global concern, particularly with the trend of over-prescribing prevalence in HICs, as evidenced by the opioid epidemic seen in some countries, including the USA (Bicket et al., 2019). In LMICs, proactive measures are essential to promote the controlled and rational use of opioids. The measures involve ensuring that patients receive appropriate opioids based on their clinical needs, with proper guidance on dosage, duration, usage, adherence, the potential harm of its misuse and regular follow-up. Such proactive measures ensure a clear understanding of appropriate prescribing practices and help avoid over-prescription. Additionally, prioritising preventive healthcare measures is paramount in tackling diseases at their onset, thereby curtailing opioid utilization in the long term. For instance, in cancer care, emphasising preventive strategies such as adopting healthy lifestyles, conducting timely screening, and vaccination not only prevents diseases (Kerschbaum & Nüssler, 2019) but also limits the reliance on opioids in future.

Given the constraints in resources and capacities to address substance use disorders, prioritising prevention, and early treatment of opioid dependency is crucial for LMICs. Furthermore, adopting an approach that emphasises the careful delivery of opioids and promotes the use of non-opioid alternatives for pain management is recommended to prevent potential opioid dependency. Additionally, research indicates that expanding coverage of opioid agonist treatment (OAT), prolonging its duration and providing OAT within prison settings can significantly reduce mortality rates and HIV-related complications associated with OUD (Degenhardt et al., 2019).

Similarly, psychosocial interventions in group settings have also shown positive benefits for family members affected by OUD. A systematic review was conducted to determine suitable psychosocial interventions so that addiction-affected families can deal with interventional methods. The methods include family psycho-educational, rational emotive behaviour therapy-based coping enhancement, intervention V and the five-step method. Except for the five-step method, which was individually delivered, these interventions were conducted in group settings across three different environments. The study also highlights the need for further research to comprehend their comprehensive benefits across diverse communities (Rane et al., 2017). The five-step methods include listening, reassuring and exploring concerns; providing targeted information; exploring coping strategies; discussing social support; and exploring additional needs (Copello et al., 2010; Velleman et al., 2011).

The review by McGovern R et al. highlights a growing recognition of the importance of addressing the well-being of family members of individuals affected by substance use (McGovern et al., 2021). Although interventions integrating substance use treatment with a family-focused approach show promise in improving family functioning and reducing conflict, there is limited evidence on the psychological well-being of affected family members. While interventions solely targeting the substance user can influence behaviour, they often fall short of providing psychological relief for family members. More interventions that directly address the psychological outcomes of affected family members are needed, alongside con-joint therapy and individual sessions for both the user and family members to address their individual needs and the interpersonal impact of substance use (McGovern et al., 2021). Long-term strategies need to be formulated and adopted to foster a culture of responsible opioid prescribing and use. Further research is necessary to develop and evaluate context-specific opioid stewardship strategies in LMICs to tackle emerging opioid-related abuse, misuse and diversion.

## Conclusion

A multifaceted approach encompassing various strategies such as capacity-building, regulatory reforms, public awareness campaigns, and international collaboration is necessary to address challenges around opioid stewardship in LMICs. Strengthening pharmacy practice, improving access to education and training for healthcare professionals, and implementing evidence-based guidelines are key to enhancing opioid stewardship in LMICs. Pharmacists in LMICs can play a crucial role by implementing diverse opioid stewardship strategies tailored to the specific context. These strategies can be built upon successful pharmacist interventions such as medication therapy adjustments, educational initiatives, policy and guideline development and risk assessment. Additionally, proactive measures focusing on prevention, early treatment, and the promotion of non-opioid alternatives for pain management are essential to curb opioid dependency.

Collaborative efforts between local governments, healthcare organisations, and international stakeholders are paramount in overcoming the barriers to opioid stewardship in LMICs. Further research is imperative to understand the local opioid epidemic, evaluate the effectiveness of interventions, and inform evidence-based policies and programmes. By prioritising opioid stewardship strategies and fostering a culture of responsible opioid use, LMICs can mitigate the risks of opioid misuse and contribute to global efforts in combating the opioid crisis.

## References

[CIT0001] ASHP statement on the pharmacist's role in substance abuse prevention, education, and assistance. (2014). *American Journal of Health-System Pharmacy*, *71*(3), 243–246. 10.2146/sp140002 24429020

[CIT0002] American Hospital Association. (2017). *Stem the tide: Addressing the opioid epidemic*. AHA. www.aha.org/opioidtoolkit

[CIT0003] Azadfard, M., Huecker, M. R., & Leaming, J. M. (2021). Opioid addiction. In *StatPearls* [Internet]. StatPearls Publishing. http://www.ncbi.nlm.nih.gov/books/NBK448203/28846246

[CIT0004] Babar, Z. U. (2021). Ten recommendations to improve pharmacy practice in low and middle-income countries (LMICs). *Journal of Pharmaceutical Policy and Practice*, *14*(1), 6. 10.1186/s40545-020-00288-2 33407945 PMC7788796

[CIT0005] Bach, P., & Hartung, D. (2019). Leveraging the role of community pharmacists in the prevention, surveillance, and treatment of opioid use disorders. *Addiction Science & Clinical Practice*, *14*(1), 30. 10.1186/s13722-019-0158-0 31474225 PMC6717996

[CIT0006] Bachhuber, M. A., Tuazon, E., Nolan, M. L., Kunins, H. V., & Paone, D. (2019). Impact of a prescription drug monitoring program use mandate on potentially problematic patterns of opioid analgesic prescriptions in New York City. *Pharmacoepidemiology and Drug Safety*, *28*(5), 734–739. 10.1002/pds.4766 30920062 PMC6689227

[CIT0007] Benyamin, R., Trescot, A. M., Datta, S., Buenaventura, R., Adlaka, R., Sehgal, N., Glaser, S. E., & Vallejo, R. (2008). Opioid complications and side effects. *Pain Physician*, *11*(2 Suppl), S105–S120. 10.36076/ppj.2008/11/S105 18443635

[CIT0008] Bicket, M. C., Brat, G. A., Hutfless, S., Wu, C. L., Nesbit, S. A., & Alexander, G. C. (2019). Optimizing opioid prescribing and pain treatment for surgery: Review and conceptual framework. *American Journal of Health-System Pharmacy*, *76*(18), 1403–1412. 10.1093/ajhp/zxz146 31505561

[CIT0009] Brown, R., & Morgan, A. (2019). The opioid epidemic in North America: Implications for Australia. *Trends and Issues in Crime and Criminal Justice*, *578*, 1–15.

[CIT0010] Bruera, E. (2018). Parenteral opioid shortage – Treating pain during the opioid-overdose epidemic. *New England Journal of Medicine*, *379*(*7*), 601–603. 10.1056/NEJMp1807117 30020849

[CIT0011] Budhathoki, S. S., Pokharel, P. K., Good, S., Limbu, S., & Bhattachan, M. (2017). Osborne RH: The potential of health literacy to address the health related UN sustainable development goal 3 (SDG3) in Nepal: A rapid review. *BMC Health Services Research*, *17*(1), 237. 10.1186/s12913-017-2183-6 28347355 PMC5369219

[CIT0012] Cernasev, A., Hohmeier, K. C., Frederick, K., Jasmin, H., & Gatwood, J. (2021). A systematic literature review of patient perspectives of barriers and facilitators to access, adherence, stigma, and persistence to treatment for substance use disorder. *Exploratory Research in Clinical and Social Pharmacy*, *2*, 100029. 35481114 10.1016/j.rcsop.2021.100029 PMC9029901

[CIT0013] Cheetham, A., Picco, L., Barnett, A., Lubman, D. I., & Nielsen, S. (2022). The impact of stigma on people with opioid use disorder, opioid treatment, and policy. *Substance Abuse and Rehabilitation*, *13*, 1–12. 10.2147/SAR.S304566 35115860 PMC8800858

[CIT0014] Chen, A., Legal, M., Shalansky, S., Mihic, T., & Su, V. (2021). Evaluating a pharmacist-led opioid stewardship initiative at an urban teaching hospital. *The Canadian Journal of Hospital Pharmacy*, *74*(3), 248–255. 34248165 10.4212/cjhp.v74i3.3152 PMC8237948

[CIT0015] Chisholm-Burns, M. A., Spivey, C. A., Sherwin, E., Wheeler, J., & Hohmeier, K. (2019). The opioid crisis: Origins, trends, policies, and the roles of pharmacists. *American Journal of Health-System Pharmacy*, *76*(7), 424–435. 10.1093/ajhp/zxy089 31361827

[CIT0016] Cid, A., Ng, A., & Ip, V. (2023). Addressing the opioid crisis-the need for a pain management intervention in community pharmacies in Canada: A narrative review. *Pharmacy (Basel)*, *11*(2). 10.3390/pharmacy11020071 PMC10144945 37104077

[CIT0017] Connery, H. S. (2015). Medication-assisted treatment of opioid use disorder: Review of the evidence and future directions. *Harvard Review of Psychiatry*, *23*(2), 63–75. 10.1097/HRP.0000000000000075 25747920

[CIT0018] Copello, A., Templeton, L., Orford, J., & Velleman, R. (2010). The 5-step method: Evidence of gains for affected family members. *Drugs: Education, Prevention and Policy*, *17*(sup1), 100–112. 10.3109/09687637.2010.514234

[CIT0019] Degenhardt, L., Grebely, J., Stone, J., Hickman, M., Vickerman, P., Marshall, B. D. L., Bruneau, J., Altice, F. L., Henderson, G., Rahimi-Movaghar, A., & Larney, S. (2019). Global patterns of opioid use and dependence: Harms to populations, interventions, and future action. *The Lancet*, *394*(10208), 1560–1579. 10.1016/S0140-6736(19)32229-9 PMC7068135 31657732

[CIT0020] Delcher, C., Pauly, N., & Moyo, P. (2020). Advances in prescription drug monitoring program research: A literature synthesis (June 2018 to December 2019). *Current Opinion in Psychiatry*, *33*(4), 326–333. 10.1097/YCO.0000000000000608 32250984 PMC7409839

[CIT0021] Dolovich, L., Austin, Z., Waite, N., Chang, F., Farrell, B., Grindrod, K., Houle, S., McCarthy, L., MacCallum, L., & Sproule, B. (2019). Pharmacy in the 21st century: Enhancing the impact of the profession of pharmacy on people's lives in the context of health care trends, evidence and policies. *Canadian Pharmacists Journal/Revue des Pharmaciens du Canada*, *152*(1), 45–53. 10.1177/1715163518815717 30719197 PMC6346332

[CIT0022] Dowell, D., Ragan, K. R., Jones, C. M., Baldwin, G. T., & Chou, R. (2022). CDC clinical practice guideline for prescribing opioids for pain – United States, 2022. *MMWR Recommendations and Reports*, *71*(3), 1–95. 10.15585/mmwr.rr7103a1 PMC9639433 36327391

[CIT0023] Drug Enforcement Administration. (2022). *DEA teams up with more than 4,300 partners to remove unneeded prescription medications from communities*. https://www.dea.gov/press-releases/2022/11/14/dea-teams-more-4300-partners-remove-unneeded-prescription-medications

[CIT0024] Egan, K. L., Gregory, E., Sparks, M., & Wolfson, M. (2017). From dispensed to disposed: Evaluating the effectiveness of disposal programs through a comparison with prescription drug monitoring program data. *The American Journal of Drug and Alcohol Abuse*, *43*(1), 69–77. 10.1080/00952990.2016.1240801 27797283

[CIT0025] Eser, N., & Çelik, N. (2022). Association between rational drug use and health literacy among pregnant women: A cross-sectional study. *Women & health*, *62*(7), 612–620. 10.1080/03630242.2022.2100033 35861057

[CIT0026] Gondora, N., Versteeg, S. G., Carter, C., Bishop, L. D., Sproule, B., Turcotte, D., Halpape, K., Beazely, M. A., Dattani, S., Kwong, M., Nissen, L., & Chang, F. (2022). The role of pharmacists in opioid stewardship: A scoping review. *Research in Social and Administrative Pharmacy*, *18*(5), 2714–2747. 10.1016/j.sapharm.2021.06.018 34261590

[CIT0027] Gregory, T., & Gregory, L. (2020). The role of pharmacists in safe opioid dispensing. *Journal of Pharmacy Practice*, *33*(6), 856–862. 10.1177/0897190019852803 31256700

[CIT0028] Harding, R., & Higginson, I. J. (2005). Palliative care in sub-Saharan Africa. *The Lancet*, *365*(9475), 1971–1977. 10.1016/S0140-6736(05)66666-4 15936427

[CIT0029] HHS. (2019). *HHS guide for clinicians on the appropriate dosage reduction or discontinuation of long-term opioid analgesics*. https://www.hhs.gov/system/files/Dosage_Reduction_Discontinuation.pdf 10.1001/jama.2019.16409 PMC7145754 31600366

[CIT0030] International Agency for Research on Cancer. (2018). *Latest global cancer data: Cancer burden rises to 18.1 million new cases and 9.6 million cancer deaths in 2018*. https://www.iarc.who.int/featured-news/latest-global-cancer-data-cancer-burden-rises-to-18-1-million-new-cases-and-9-6-million-cancer-deaths-in-2018.

[CIT0031] Kerschbaum, E., & Nüssler, V. (2019). Cancer prevention with nutrition and lifestyle. *Visceral Medicine*, *35*(4), 204–209. 10.1159/000501776 31602380 PMC6738231

[CIT0032] Knapp, C., Woodworth, L., Wright, M., Downing, J., Drake, R., Fowler-Kerry, S., Hain, R., & Marston, J. (2011). Pediatric palliative care provision around the world: A systematic review. *Pediatric Blood & Cancer*, *57*(3), 361–368. 10.1002/pbc.23100 21416582

[CIT0033] Kolodny, A., Courtwright, D. T., Hwang, C. S., Kreiner, P., Eadie, J. L., Clark, T. W., & Alexander, G. C. (2015). The prescription opioid and heroin crisis: A public health approach to an epidemic of addiction. *Annual Review of Public Health*, *36*(1), 559–574. 10.1146/annurev-publhealth-031914-122957 25581144

[CIT0034] Krashin, D., Trescot, A., & Murinova, N. (2013). Opioids and pain treatment. In K. G. Ramawat & J.-M. Mérillon (Eds.), *Natural products: Phytochemistry, botany and metabolism of Alkaloids, phenolics and terpenes* (pp. 1367–1382). Springer Berlin Heidelberg.

[CIT0035] McCabe, S. E., West, B. T., Veliz, P., McCabe, V. V., Stoddard, S. A., & Boyd, C. J. (2017). Trends in medical and nonmedical use of prescription opioids among US adolescents: 1976–2015. *Pediatrics*, *139*(4). 10.1542/peds.2016-2387 PMC5369669 28320868

[CIT0036] McGovern, R., Smart, D., Alderson, H., Araújo-Soares, V., Brown, J., Buykx, P., Evans, V., Fleming, K., Hickman, M., Macleod, J., Meier, P., & Kaner, E. (2021). Psychosocial interventions to improve psychological, social and physical wellbeing in family members affected by an adult relative's substance use: A systematic search and review of the evidence. *International Journal of Environmental Research and Public Health*, *18*(4), 1793. 10.3390/ijerph18041793 33673199 PMC7918716

[CIT0037] Miller, R., & Goodman, C. (2016). Performance of retail pharmacies in low- and middle-income Asian settings: A systematic review. *Health Policy and Planning*, *31*(7), 940–953. 10.1093/heapol/czw007 26962123 PMC4977427

[CIT0038] Nahin, R. L., Boineau, R., Khalsa, P. S., Stussman, B. J., & Weber, W. J. (2016). Evidence-based evaluation of complementary health approaches for pain management in the United States. *Mayo Clinic Proceedings*, *91*(9), 1292–1306. 10.1016/j.mayocp.2016.06.007 27594189 PMC5032142

[CIT0039] National Academies of Sciences, Engineering, and Medicine; Health and Medicine Division; Board on Health Sciences Policy; Committee on Pain Management and Regulatory Strategies to Address Prescription Opioid Abuse. (2017). Pain management and the opioid epidemic: Balancing societal and individual benefits and risks of prescription opioid use. https://pubmed.ncbi.nlm.nih.gov/29023083/29023083

[CIT0040] National Institute on Drug Abuse. (2023). *Drug overdoes death rates*. https://nida.nih.gov/research-topics/trends-statistics/overdose-death-rates

[CIT0041] NEJM Knowledge+ Team. (2020). *Non-opioid analgesics role in pain management*. NEJM Knowledge+. https://knowledgeplus.nejm.org/blog/non-opioid-analgesics-role-in-pain-management/

[CIT0042] Noncommunicable diseases. (2023). *World Health Organization*. https://www.who.int/news-room/fact-sheets/detail/noncommunicable-diseases

[CIT0043] Norn, S., Kruse, P. R., & Kruse, E. (2005). History of opium poppy and morphine. *Dansk Medicinhistorisk Arbog*, *33*, 171–184. 17152761

[CIT0044] Opioid Use Disorder. (2024). *Johns Hopkins Medicine*. https://www.hopkinsmedicine.org/health/conditions-and-diseases/opioid-use-disorder

[CIT0045] Pathan, H., & Williams, J. (2012). Basic opioid pharmacology: An update. *British Journal of Pain*, *6*(1), 11–16. 10.1177/2049463712438493 26516461 PMC4590096

[CIT0046] Popovici, I., Maclean, J. C., Hijazi, B., & Radakrishnan, S. (2018). The effect of state laws designed to prevent nonmedical prescription opioid use on overdose deaths and treatment. *Health Economics*, *27*(2), 294–305. 10.1002/hec.3548 28719096

[CIT0047] Rane, A., Church, S., Bhatia, U., Orford, J., Velleman, R., & Nadkarni, A. (2017). Psychosocial interventions for addiction-affected families in low and middle income countries: A systematic review. *Addictive Behaviors*, *74*, 1–8. 10.1016/j.addbeh.2017.05.015 28554034

[CIT0048] Rosen, Y. (2023). Opioid education program for grades 6 to 12 considered by Alaska lawmakers. *Alaska Beacon*, March 6*.* https://alaskabeacon.com/briefs/opioid-education-program-for-grades-6-to-12-considered-by-state-lawmakers/

[CIT0049] Rosenblum, A., Marsch, L. A., Joseph, H., & Portenoy, R. K. (2008). Opioids and the treatment of chronic pain: Controversies, current status, and future directions. *Experimental and Clinical Psychopharmacology*, *16*(5), 405–416. 10.1037/a0013628 18837637 PMC2711509

[CIT0050] Rural Health Information Hub. (2020). *Proper drug disposal programs*. https://www.ruralhealthinfo.org/toolkits/substance-abuse/2/harm-reduction/drug-disposal

[CIT0051] Sapkota, B., Giri, A., Bhatta, B., Awasthi, K., Bhurtyal, K., Joshi, B., & Joshi, K. R. (2022). Implementation of medicine take-back concept at community level in Nepal: A pilot study. *Journal of Public Health*, *44*(3), 575–585. 10.1093/pubmed/fdab134 33912964

[CIT0052] Sasaki, H., Bouesseau, M. C., Marston, J., & Mori, R. (2017). A scoping review of palliative care for children in low- and middle-income countries. *BMC Palliative Care*, *16*(1), 60. 10.1186/s12904-017-0242-8 29178866 PMC5702244

[CIT0053] Schwartz, R. P., Gryczynski, J., O'Grady, K. E., Sharfstein, J. M., Warren, G., Olsen, Y., Mitchell, S. G., & Jaffe, J. H. (2013). Opioid agonist treatments and heroin overdose deaths in Baltimore, Maryland, 1995–2009. *American Journal of Public Health*, *103*(5), 917–922. 10.2105/AJPH.2012.301049 23488511 PMC3670653

[CIT0054] Shrestha, R., & Shrestha, S. (2024). Addressing the critical gap: Ensuring urgent access to palliative care services with essential medications in Nepal. *Journal of Pain & Palliative Care Pharmacotherapy*, 1–10. 10.1080/15360288.2024.2320384 38441942

[CIT0055] Shrestha, S., Iqbal, A., Teoh, S. L., Khanal, S., Gan, S. H., Lee, S. W. H., & Paudyal, V. (2024). Impact of pharmacist-delivered interventions on pain-related outcomes: An umbrella review of systematic reviews and meta-analyses. * Research in Social and Administrative Pharmacy*, S1551-7411(24)00091-3. 10.1016/j.sapharm.2024.03.005 38514293

[CIT0056] Shrestha, S., Kc, B., Blebil, A. Q., & Teoh, S. L. (2022). Pharmacist involvement in cancer pain management: A systematic review and meta-analysis. *The Journal of Pain*, *23*(7), 1123–1142. 10.1016/j.jpain.2022.02.002 35151871

[CIT0057] Shrestha, S., Khatiwada, A. P., Gyawali, S., Shankar, P. R., & Palaian, S. (2020). Overview, challenges and future prospects of drug information services in nepal: A reflective commentary. *Journal of Multidisciplinary Healthcare*, *13*, 287–295. 10.2147/JMDH.S238262 32256077 PMC7090186

[CIT0058] Shrestha, S., Khatiwada, A. P., Sapkota, B., Sapkota, S., Poudel, P., Kc, B., Teoh, S. L., Blebil, A. Q., & Paudyal, V. (2023). What is "opioid stewardship"? An overview of current definitions and proposal for a universally acceptable definition. *Journal of Pain Research*, *16*, 383–394. 10.2147/JPR.S389358 36798077 PMC9926985

[CIT0059] Smith, R. G. (2020). A process review to an interdisciplinary approach to opioid stewardship. *Journal of Interprofessional Education & Practice*, *20*, 100344. 10.1016/j.xjep.2020.100344

[CIT0060] Soltani, H., & Pardakhty, A. (2016). Marketed new drug delivery systems for opioid agonists/antagonists administration: A rapid overview. *Addiction & Health*, *8*(2), 115–122. 27882209 PMC5115645

[CIT0061] Thakur, T., Frey, M., & Chewning, B. (2021). Communication between patients and health care professionals about opioid medications. *Exploratory Research in Clinical and Social Pharmacy*, *2*, 100030. 35481112 10.1016/j.rcsop.2021.100030 PMC9030717

[CIT0062] Thapa, P., Lee, S. W. H., KC, B., Dujaili, J. A., Mohamed Ibrahim, M. I., & Gyawali, S. (2021). Pharmacist-led intervention on chronic pain management: A systematic review and meta-analysis. *British Journal of Clinical Pharmacology*, *87*(8), 3028–3042. 10.1111/bcp.14745 33486825

[CIT0063] The Frontline in the Fight Against Addiction. (2024). https://searidgedrugrehab.com/article/pharmacies-recovery/

[CIT0064] Trang, T., Al-Hasani, R., Salvemini, D., Salter, M. W., Gutstein, H., & Cahill, C. M. (2015). Pain and poppies: The good, the bad, and the ugly of opioid analgesics. *The Journal of Neuroscience*, *35*(41), 13879–13888. 10.1523/JNEUROSCI.2711-15.2015 26468188 PMC4604226

[CIT0065] UNODC. (2023). Drivers of Illicit Trafficking in Border Communities in Southeast Asia.

[CIT0066] Uritsky, T. J., Busch, M. E., Chae, S. G., & Genord, C. (2021). *Opioid stewardship: Building on antibiotic stewardship principles* (Vol. 34, pp. 181–183). Taylor & Francis. 10.1080/15360288.2020.1765066 32757904

[CIT0067] Veettil, S. K., Darouiche, G., Sawangjit, R., Cox, N., Lai, N. M., & Chaiyakunapruk, N. (2022). Effects of pharmacist interventions on pain intensity: Systematic review and meta-analysis of randomized controlled trials. *Journal of the American Pharmacists Association*, *62*(4), 1313–1320.e1316. 10.1016/j.japh.2022.02.015 35307311

[CIT0068] Velleman, R., Orford, J., Templeton, L., Copello, A., Patel, A., Moore, L., Macleod, J., & Godfrey, C. (2011). 12-month follow-up after brief interventions in primary care for family members affected by the substance misuse problem of a close relative. *Addiction Research & Theory*, *19*(4), 362–374. 10.3109/16066359.2011.564691

[CIT0069] Vowles, K. E., McEntee, M. L., Julnes, P. S., Frohe, T., Ney, J. P., & van der Goes, D. N. (2015). Rates of opioid misuse, abuse, and addiction in chronic pain: A systematic review and data synthesis. *Pain*, *156*(4), 569–576. 10.1097/01.j.pain.0000460357.01998.f1 25785523

[CIT0070] Webb, K., Cernasev, A., Li, M. S., Gatwood, J., Cochran, G., & Hohmeier, K. C. (2021). An exploratory study of pharmacist perceptions of opioid interventions for acute pain. *Journal of Pharmacy Technology*, *37*(1), 36–44. 10.1177/8755122520967766 PMC7809323 34753156

[CIT0071] WHO. (2023). *Opioid overdose*. https://www.who.int/news-room/fact-sheets/detail/opioid-overdose

[CIT0072] Wu, P. E., Lee, T. C., & McDonald, E. G. (2023). What should i know about medication deprescribing? *JAMA Internal Medicine*, *183*(8), 891–891. 10.1001/jamainternmed.2023.2099 37273220

